# Beside Resting-State EEG in the Diagnosis and Prognosis of Patients with Disorders of Consciousness

**DOI:** 10.3390/diagnostics16172801

**Published:** 2026-08-31

**Authors:** Li Yang, Yejing Sun, Wentao Zeng, Xiangqiang Meng, Pei He, Zhen Feng, Yang Bai

**Affiliations:** 1 Nanchang University Affiliated Rehabilitation Hospital, Nanchang 330003, China; yangli_ncu@163.com (L.Y.); m13767170744@163.com (Y.S.); wentaozeng@aliyun.com (W.Z.); mengxiangqiang2023@163.com (X.M.); 2 Rehabilitation Medicine Clinical Research Center of Jiangxi Province, Nanchang 330006, China; 3 Jiangxi Provincial Health Commission Key Laboratory for Rehabilitation of Consciousness Disorders, Nanchang 330006, China; 4 Jiangxi Provincial Women’s and Children’s Medical Center, Nanchang 330038, China; 5 The First Affiliated Hospital of Nanchang University, Nanchang 330006, China; hepei19930814@163.com

**Keywords:** resting-state EEG, disorders of consciousness, behavioral assessment, permutation entropy, weighted Phase Lag Index

## Abstract

**Background/Objectives**: Behavioral scales are the primary diagnostic tool for disorders of consciousness (DOC) but carry a high risk of misdiagnosis. Bedside resting-state EEG (rs-EEG) enables non-invasive objective brain function evaluation, although its diagnostic and prognostic value for DOC remains understudied. **Methods**: This study enrolled 207 DOC patients with guardian-signed informed consent. Weekly bedside 4-channel rs-EEG and CRS-R behavioral assessments were conducted during hospitalization. We systematically compared three categories of EEG metrics: nonlinear entropies (PE, SamEn), phase coupling parameters, and functional connectivity indices (SI, wPLI), evaluating their ability to stratify consciousness and forecast outcomes. **Results**: Permutation entropy (PE) stood out with triple functions: cross-sectional consciousness grading, longitudinal dynamic tracking, and prognosis prediction, surpassing all other EEG features. SamEn merely discriminates VS/UWS from MCS/EMCS but fails to separate MCS and EMCS. Power spectra, phase coupling, SI, and wPLI only yield group discrepancies at limited leads or frequency bands with unstable stratification, acting merely as auxiliary markers. Subtype-specific prognostic signatures were identified: frontal β-wPLI and parietal PE predict consciousness recovery in VS/UWS, whereas fronto−parietal α-wPLI predicts improvement in MCS patients. **Conclusions**: The bedside rs-EEG reliably assesses consciousness and predicts DOC outcomes, and PE possesses prominent clinical value for DOC diagnosis and prognostic stratification.

## 1. Introduction

In clinical neurorehabilitation, consciousness is defined as the integrated capacity for wakefulness and awareness of self and the environment, which is evaluated based on standardized behavioral responses. Disorders of consciousness (DOC) are clinical conditions characterized by impaired awareness following severe brain or neurological injury [1,2,3]. DOC primarily includes Vegetative State/Unresponsive Wakefulness Syndrome (VS/UWS) and Minimally Conscious State (MCS) [4]. Early post-mortem and neuroimaging investigations have investigated anatomical substrates underlying VS/UWS and MCS [5]. These two clinical states do not correspond to injuries confined to completely separate brain regions. Instead, they share largely overlapping vulnerable structures including the thalamus, widespread white-matter pathways, and frontoparietal associative cortices. VS/UWS is characterized by more severe, diffuse disruption of thalamo-cortical networks. MCS occurs when partial structural and functional integrity of these large-scale circuits is retained, allowing minimal behavioral evidence of awareness despite substantial residual brain damage [6].The clinical distinctions between the two are as follows: VS/UWS: Patients exhibit complete sleep−wake cycles, characterized by periodic changes between eye-open and eye-closed states, yet show no signs of self-awareness or environmental awareness. They may display purposeless eye movements, moans, or facial expressions, but these behaviors are reflexive rather than meaningful responses to stimuli. Patients cannot follow commands, perform purposeful movements, produce intelligible speech or communicate in any form [7,8]; MCS: Patients exhibit distinct but fluctuating signs of consciousness and demonstrate limited yet clear awareness of themselves or their environment. Diagnosis of MCS requires at least one of the following behaviors: following simple commands (e.g., “open your eyes”, “hold my hand”), giving yes/no responses (regardless of accuracy), producing intelligible speech, demonstrating orienting responses to stimuli (e.g., eye-tracking moving objects), or exhibiting context-appropriate emotional reactions such as smiling or crying (as opposed to reflexive expressions). Although MCS patients exhibit a higher level of consciousness than VS/UWS individuals, they remain unable to engage in functional communication or independent activities of daily living [9]. Correctly categorizing the diagnosis of DOC is critical for formulating clinical management strategies. This is not only because MCS patients typically have a better prognosis than VS/UWS patients [10,11,12], but also because patient outcomes are also modulated by multiple individual factors, such as age at the time of acute brain injury and the underlying cause. However, the diagnosis and prognosis of DOC have been difficult problems for neuroscientists and clinicians due to the diversity of consciousness manifestations [13]. Currently, the Coma Recovery Scale-Revised (CRS-R) remains the most accepted and recommended tool for assessing the level of consciousness [14,15]. However, relying solely on the scale to assess the level of consciousness may result in misdiagnosis, limited by the operator’s proficiency and the patient’s cooperation during the assessment [16]. Therefore, the most pressing challenge at present is to identify more objective, convenient, and scientifically sound methods for assessing the level of consciousness in DOC patients and evaluating their prognosis.

Electroencephalography (EEG) recording is a widely applicable, cost-effective, and feasible neurophysiological technique for evaluating patients with DOC [17]. Neural signals in the brain exhibit non-stationarity, undergoing dynamic transitions between distinct functional states, even during resting periods [18,19,20,21]. Therefore, the choice of resting-state electroencephalography (rs-EEG) for monitoring DOC patients has proven to be a more practical option. The EEG features utilized in studies on DOC are commonly divided into three categories: spectral features, nonlinear features, and functional connectivity features [22]. The most commonly utilized features for discerning the consciousness levels of patients with DOC and for predicting their future outcomes are spectral power, entropy, and coherence analyses [22,23]. The power spectral density (PSD) in the δ and θ bands was positively correlated with the prognostic level of DOC patients [24]. The relative band power (RBP) can differentiate healthy individuals from patients with DOC [23]. The quadratic phase coupling (QPC) of neural oscillations is also manifested through a specific spectral feature [25]. Permutation Entropy (PE) and Sample Entropy (SamEn) are commonly utilized techniques for assessing the complexity of EEG signals [23].The synchronization index (SI) is indicative of the rhythmicity and complexity of EEG activity within the cerebral cortex [26]. As a critical functional connectivity indicator, the weighted Phase Lag Index (wPLI) can reflect the severity of brain injury and possesses favorable predictive value for long-term prognosis in DOC patients [27]. From a physiological perspective, consciousness during wakefulness is primarily dominated by β and γ oscillations; the α rhythm is characteristic of wakefulness in a relaxed state, while high-amplitude δ slow waves dominate deep sleep. In states of impaired consciousness, brain activity shifts toward slower rhythms. Nevertheless, EEG signals still reflect a mixed state of general arousal levels and consciousness-related neural processing. Since different frequency bands play distinct functional roles, spectral characteristics alone cannot fully distinguish changes in subjective consciousness from changes in arousal levels. Measures of nonlinear complexity and functional connectivity may provide complementary information for assessing consciousness-related brain functions. In summary, EEG data can be delineated across diverse dimensions through various analytical approaches, with multidimensional rs-EEG characteristics serving as a crucial foundation for evaluating consciousness levels [28]. Nevertheless, conventional standard EEG examinations require patient transfer to dedicated EEG rooms, which is impractical for critically ill or immobile DOC patients. In addition, cranial defects commonly observed in DOC patients impede high-quality whole-brain EEG recording, prolong data acquisition, and ultimately generate massive but heterogeneous raw datasets.

In recent years, with advances in technology, bedside electroencephalography has emerged as a tool for monitoring brain function, suitable for patients with impaired consciousness and critically ill patients with limited mobility [29,30]. From a physiological perspective, consciousness during wakefulness is primarily dominated by β (13–30 Hz) and γ oscillations (30–40 Hz); the α rhythm (8–13 Hz) is characteristic of relaxed wakefulness, while high-amplitude δ slow waves (1–4 Hz) dominate deep sleep. In states of impaired consciousness, brain activity shifts toward slower rhythms. Nevertheless, EEG signals still reflect a mixed state of general arousal levels and neural processing related to consciousness. Since different frequency bands play distinct functional roles, spectral characteristics alone cannot fully distinguish between changes in subjective consciousness and changes in arousal levels. Measures of nonlinear complexity and functional connectivity may provide supplementary information for assessing brain functions related to consciousness. The portability and real-time monitoring capabilities of bedside EEG render it potentially an ideal instrument for evaluating brain function in patients with DOC in rehabilitation wards [31]. Most bedside EEG studies of DOC are cross-sectional comparisons between individuals, but due to high individual variability, misdiagnosis can easily occur. Longitudinal comparisons of EEG characteristics can be effective in reducing patient misdiagnosis and improving patient management. Utilizing the longitudinal characteristics of bedside EEG monitoring data is key to detecting fluctuations in consciousness. However, the selection of bedside rs-EEG features for classification, diagnosis, and prognostic prediction of DOC remains unclear [32,33].

In this study, we attempted to narrow down the individual differences by comparing the rs-EEG features of patients with different levels of consciousness horizontally and comparing the rs-EEG features of the same patient with different levels of consciousness vertically, so as to screen out the bedside EEG features that are suitable for the diagnosis of DOC and the assessment of clinical prognosis.

## 2. Materials and Methods

### 2.1. Patients

All participants in this study completed the informed consent process; since the study population consisted of patients with DOC, their legal guardians signed the written informed consent forms on their behalf. This study was approved by the Ethics Committee of the First Affiliated Hospital of Nanchang University (Approval No.: IIT2023-222). This study complied with the Declaration of Helsinki and relevant ethical guidelines. This study enrolled patients with disorders of consciousness (DOC) admitted to the Department of Rehabilitation Medicine at the First Affiliated Hospital of Nanchang University between September 2021 and September 2023. A total of 207 patients were included. During hospitalization, routine bedside resting-state electroencephalography (rs-EEG) monitoring and Coma Recovery Scale-Revised (CRS-R) assessments were performed weekly. Both procedures were part of the standard clinical care protocol for patients with DOC in our department, with data systematically accumulated throughout routine clinical practice. The study protocol was designed retrospectively following data collection, allowing for the unified organization, analysis, and interpretation of existing clinical data. Exclusion criteria were as follows: a history of neurological or psychiatric disorders; use of sedative medications within 24 h prior to EEG monitoring; significant artifacts during EEG examination; death within 24 h of admission; discharge against medical advice; or transfer to another department.

Basic demographic information, including age, sex, etiology, and disease duration, was collected for all patients. Subsequently, all enrolled patients followed the standardized DOC rehabilitation clinical pathway established by our department.

### 2.2. Behavioral Assessment

The assessment of consciousness levels in DOC is dependent on the CRS-R scale [34], which assesses the patient’s response to a set of hierarchically ranked items in six different domains: auditory, visual, motor, verbal, communication, and arousal. Assessments are conducted once per week, three times per day. Each CRS-R assessment was performed by two specially trained physicians three times a day, and the final assessment was based on the diagnosis of the highest level of consciousness among the three times.

This study utilized a comparison between the initial CRS-R and the final CRS-R (the last assessment before discharge) during the patient’s hospitalization to observe the patient’s recovery of consciousness [35].

### 2.3. Patients Group

Patients with DOC were divided into two major groups based on their initial and final CRS-R scores during hospitalization: the unimproved consciousness group and the improved consciousness group. The unimproved group was subdivided into two categories: VS/UWS and MCS. Statistical mapping also included EMCS patients in the comparison since the emergence from the minimally conscious state (EMCS) group was intermediate between the DOC and normal groups and significantly different from MCS and VS/UWS in terms of brain function, which was informative, but excluded EMCS patients from the unimproved group since they were not part of the DOC. The consciousness improvement group was divided into three subgroups according to the degree of improvement: MCS (recovery from VS/UWS), EMCS (recovery from VS/UWS), and EMCS (recovery from MCS).

### 2.4. Data Acquisition and Preprocessing

The portable EEG equipment used at the bedside was a brain function monitor (Nicolet Monitor) produced by Natus Neurology Incorporated (3150 Pleasant View Road, Middleton, WI, USA; IDA Business Park, Gort, Co., Galway, Ireland). Stimulants, tranquilizers, and antiepileptic drugs were strictly prohibited 24 h prior to the EEG. Patients were instructed to wash their hair on the day before the test. To minimize distractions during the test, patients were usually tested when the room was quiet, while family members kept electronic devices turned off during the evaluation and remained as quiet as possible except for necessary daily activities. Scalp electrodes were positioned based on the International 10–20 System, with placements in the left and right frontal regions (F3, F4) as well as the left and right parietal regions (P3, P4). The mid-frontal point (Fz) was grounded, with the reference electrode linked to a spot situated 1 cm behind the central midline point (Cz). The data were recorded at a sampling frequency of 125 Hz, and the impedance of the electrodes remained below 5 kΩ during the entire acquisition process.

Weekly CRS-R assessments were performed on all hospitalized patients with disorders of consciousness. To determine the current level of consciousness, each weekly assessment consisted of at least three evaluations conducted by two trained specialists. Following the CRS-R assessment, bedside EEG monitoring was performed for approximately 1 to 3 h. This assessment protocol was repeated weekly throughout the patient’s hospitalization. Patients were subsequently grouped based on the improvement in their CRS-R scores as follows: For patients showing no change in consciousness level, only one EEG dataset corresponding to a single consciousness state was extracted. For patients exhibiting changes in consciousness level, separate EEG datasets were extracted and analyzed for each distinct consciousness state.

The EEG data were preprocessed using the EEGLAB v2023.0 [36] toolbox within MATLAB R2018b. The researchers then manually screened the extracted EEG data, selecting half-hourly data with fewer artifacts and interferences as the initial dataset. Then, the half-hourly dataset was divided into six independent segments and imported into EEGLAB v2023.0, with each segment being 5 min long and then divided into 10 s intervals. Subsequently, EEG data in the range of 1 Hz to 40 Hz were filtered and averaged with reference to the mean. Artifactual segments were manually removed. These included the following: (1) blink artifacts, identified based on characteristic waveforms in frontal channels and amplitude thresholds; (2) myoelectric artifacts, identified based on high-frequency activity and distinct waveform morphology; (3) movement artifacts, identified by simultaneous, abrupt fluctuations across multiple channels; and (4) channel noise, identified by persistent signal loss or abnormal impedance within specific channels (performed independently by two researchers to ensure consistency).

### 2.5. Feature Extraction

#### 2.5.1. Spectral Characteristics

Power Spectral Density (PSD)

Power spectral density (PSD) was computed using Welch’s method, a technique based on the Fourier transform [37]. PSD estimates the power distribution of EEG signals across different frequencies. The power spectrum was segmented into multiple frequency bands ranging from 0.1 to 45 Hz to calculate relative power. Power values were averaged within each conventional frequency band (δ, θ, α, β, and γ) and subsequently averaged across all epochs.

Relative Band Power (RBP)

The RBP, a prevalent analytical technique in EEG analysis, assesses the energy of different frequency components within EEG signals. RBP was determined across five frequency bands: δ (1–4 Hz), θ (4–8 Hz), α (8–13 Hz), β (13–30 Hz), and γ (30–40 Hz). The relative power of each given band was calculated by

(1) $$\mathrm{Relative}\;\mathrm{power}\left(f1,f2\right)=\frac{\mathrm{Power}\left(f1,f2\right)}{\mathrm{Power}\left(1,45\right)}\times 100\%$$

where $\mathrm{power}\left(f1,f2\right)$ indicates the power between low f1 and high f2 frequencies estimated by the periodogram method. $\mathrm{power}\left(1,45\right)$ means the sum of power (1–45 Hz).

Quadratic Phase Coupling (QPC)/Quadratic Phase Self-Coupling (QPSC)

The key difference between QPC and QPSC lies in whether the frequency components involved in the coupling are the same. QPC involves two different fundamental frequencies. QPSC is a special case of QPC, in which the two frequencies involved in the coupling are identical. QPC in EEG signals was quantified using wavelet bicoherence based on the general harmonic wavelet transform (GHWT) combined with a segment-averaging approach [25,38]. This method, referred to as QPSC, enables the detection of nonlinear interactions within EEG signals by identifying phase-locked relationships (i.e., quadratic phase coupling) between different frequency components, thereby reflecting nonlinear integration mechanisms within neuronal ensembles. Bicoherence was computed on wavelet coefficients $a\left(f,t\right)$ in all pairs of frequencies from 1 to 40 Hz, with a step of 1 Hz and a bandwidth of 1 Hz. Firstly, GHWT was conducted at the EEG epoch $X_{k}\left(t\right)$ (k means the number of epochs), obtaining the wavelet coefficients $a_{k}\left(f_{p},t\right)$ corresponding to the signal component at frequency $f_{p}$ . In order to reduce edge effects, we padded data with zeros to get N up to the next higher power of two and removed the corresponding time points after transform. Then, the normalized squared wavelet bicoherence, which characterizes the QPC between component with frequencies $f_{p}$ and $f_{q}$ in $X_{k}\left(t\right)$ , is calculated as

(2) $$b_{k}\left(f_{p},f_{q}\right)=\frac{{\left|B_{k}\left(f_{p},f_{q}\right)\right|}^{2}}{\sum_{t=1}^{N}|a_{k}\left(f_{p},t\right)a_{k}(f_{q},t){|}^{2}\sum_{t=1}^{N}|a_{k}(f_{p}+f_{q},t){|}^{2}}$$

where the phase randomized wavelet bi-spectrum is given by the expression

(3) $$B_{k}\left(f_{p},f_{q}\right)=\sum_{t=1}^{N}a_{k}\left(f_{p},t\right)a_{k}\left(f_{q},t\right)a_{k}\times \left(f_{p}+f_{q},t\right)e^{\mathrm{iR}\emptyset_{k\left(f_{p},f_{q},t\right)}}$$

$R\in \left(-\pi ,\pi \right]$ is a random variable and the biphase is

(4) $$\emptyset_{k}\left(f_{p},f_{q},t\right)=\emptyset_{k}\left(f_{p},t\right)+\emptyset_{k}\left(f_{q},t\right)-\emptyset_{k}\left(f_{p}+f_{q},t\right)$$

Then, we use a surrogate method to correct the false coupling. Using a new bi-phase

(5) $$\emptyset_{k}^{\#}\left(f_{p},f_{q},t\right)=\emptyset_{k}\left(f_{p},f_{q},t\right)+\theta$$

to replace $\emptyset_{k}\left(f_{p,}f_{q},t\right)$ , $\theta \in \left(-\pi ,\pi \right]$ , a new surrogated bicoherence value can be obtained. After surrogating over $N_{\mathrm{sum}}$ times ( $N_{\mathrm{sum}}$ > 30), bicoherence values greater than the 95% statistical threshold (mean plus 1.96 times standard deviation) are preserved, and the other is set to zero. Finally, for all the epochs, Q-test is applied to remove the outlier bicoherence values to obtain a reliable bicoherence value. Then, a symmetrical bicoherence matrix could be obtained, with each element $b\left(f_{p,}f_{q}\right)$ , denoting the bicoherence value at a bifrequency pair $\left(f_{p,}f_{q}\right)$ , where 1 ≤ $\left(f_{p,}f_{q}\right)$ ≤ 45 Hz. The diagonal line values of the bicoherence matrix reveal QPSC. Averaged diagonal line values within 1–4 Hz, 4–8 Hz, and 8–13 Hz represent δ-QPSC, θ-QPSC, and α-QPSC, respectively.

#### 2.5.2. Nonlinear Features

Permutation Entropy (PE)

The PE is a measure of complexity proposed by Bandt and Pompe for chaotic time series, in particular, in the presence of dynamic and observational noise [39]. In this study, we utilized the perms function in MATLAB for the generation of permutations. The classic parameter settings were used when calculating the PE: The embedding dimension was set to 3, and the time delay was set to 1. The parameter selections for PE (m = 3, τ = 1) followed established conventions in the DOC and EEG literature [40]. A lower embedding dimension was chosen because longer dimensions would require exponentially more data points to reliably estimate ordinal pattern probabilities, particularly challenging given the reduced signal complexity in DOC patients. With a sampling rate of 125 Hz, an embedding dimension of m = 3 and a time delay of τ = 1 yield a pattern window of 24 ms, which is sufficient to resolve neural oscillations up to 40 Hz. The perms function accepts a vector of length n , generates all permutations of n elements, utilizes the count vector to determine the probability of each permutation and subsequently computes the entropy of the sequence of permutations. Calculate the probability that each permutation occurs in each order based on the number of times it occurs. The PE was calculated by

(6) $$\mathrm{PE}=-\sum_{j=1}^{n!}p_{j}\ln(p_{j})$$

Sample Entropy (SamEn)

The rise in SamEn is commonly correlated with an increase in complexity. The computation of SamEn was conducted on the raw EEG data from all subjects at each electrode. The calculation of SamEn is as follows: Arrange x (1), x (2), …, x (N) to form an m-dimensional vector. For SamEn, we used an embedding dimension of m = 2 and a tolerance threshold of r = 0.2 × SD, following standard physiological time-series analysis [41]. This setting ensures noise robustness while preserving signal complexity: m = 2 minimizes data requirements and r = 0.2 × SD suppresses noise without masking fine structures. The time delay was τ = 1 (consecutive samples), which at 125 Hz yields an 8 ms resolution adequate for capturing 1–40 Hz neural dynamics.

(7) $$X_{m\left(i\right)}=\left[x\left(i\right),x\left(i+1\right),\ldots ,x\left(i+m-1\right)\right];1\leq i\leq N-m+1$$

Define $d\left[X_{m}\left(i\right),X_{m}\left(j\right)\right]$ as the largest distance between $X_{m}\left(i\right)$ and $X_{m}\left(j\right)$ :

(8) $$d\left[X_{m}\left(i\right),X_{m}\left(j\right)\right]=\max\left|x\left(i+k\right)-x\left(j+k\right)\right|$$

where 1 ≤ k ≤ m − 1, 1 ≤ i , j ≤ N−m−1 , i≠j . Given a threshold value r (r > 0), for 1≤i≤N−m, i≠j , define $B_{i}^{m}\left(r\right)$ as:

(9) $$B_{i}^{m}\left(r\right)=\frac{1}{N-m-1}\mathrm{num}\left\{d\left[X_{m}\left(i\right),X_{m}\left(j\right)\right]<r\right\}$$

We can calculate the average for all of i :

(10) $$B^{m}\left(r\right)=\frac{1}{N-m-1}\overset{N-m}{\underset{i=1}{\sum }}B_{i}^{m}\left(r\right)$$

For m + 1, we have the following:

(11) $$A_{i}^{m}\left(r\right)=\frac{1}{N-m-1}\mathrm{num}\left\{d\left[X_{m+1}\left(i\right),X_{m+1}\left(j\right)\right]<r\right\}$$

where 1≤i≤N−m, i≠j . The average for all of i is shown as follows:

(12) $$A^{m}\left(r\right)=\frac{1}{N-m-1}\overset{N-m}{\underset{i=1}{\sum }}A_{i}^{m}\left(r\right)$$

SamEn can be calculated as:

(13) SamEnm,r=limN→∞−lnAmrBmr

Synchronization index (SI)

The SI is a quantitative nonlinear metric widely applied to characterize interregional neural coupling and oscillatory synchronization dynamics based on cross-frequency phase-amplitude coupling mechanisms. This index quantifies the overall intensity of functional synchronization between distinct brain regions by evaluating statistical interdependencies of multi-channel EEG oscillatory signals across time series. Higher SI values indicate stronger phase consistency, more stable neural coupling, and higher functional integration of large-scale cortical networks, whereas lower SI values reflect desynchronized neural activity and weakened interregional functional cooperation. In the present study, the SI was calculated based on phase–amplitude coupling analysis between low-frequency phase and high-frequency amplitude fluctuations. The SI was calculated by

(14) $$\mathrm{SI}=\frac{1}{n}\times \overset{n}{\underset{t=1}{\sum }}e^{i}\left[\emptyset_{\mathrm{lt}}-\emptyset_{\mathrm{ut}}\right]$$

where n denotes the total number of time points in the EEG data, t denotes the time point, $\emptyset_{\mathrm{lt}}$ denotes the phase of the low-frequency band EEG at time t, and $\emptyset_{\mathrm{ut}}$ denotes the phase of the power time series of the high-frequency band EEG at time t. The absolute value of SI ranges from 0 to 1. Specifically, an SI value approaching 1 indicates highly stable cross-frequency synchronization between paired brain regions, while a value close to 0 suggests random, unsynchronized neural oscillations with negligible functional coupling. This standardized calculation procedure was uniformly applied to all participants to guarantee consistent, reproducible, and comparable quantitative results across the entire dataset.

#### 2.5.3. Functional Connectivity

weighted Phase Lag Index (wPLI)

The weighted Phase Lag Index (wPLI) is a robust phase-based functional connectivity metric that quantifies phase synchronization between paired EEG signals while effectively minimizing the confounding effects of volume conduction and zero-lag artifacts. Compared with conventional coherence and Phase Lag Index (PLI), wPLI assigns adaptive weights according to the magnitude of imaginary cross-spectral components, thereby improving the reliability and sensitivity of detecting genuine inter-regional neural interactions. In this study, EEG signals were first decomposed into five canonical frequency bands: δ (1–4 Hz), θ (4–8 Hz), α (8–13 Hz), β (13–30 Hz), and γ (30–45 Hz). For each predefined frequency band, the analytic signals of all artifact-free EEG channels were extracted via Hilbert transformation. The wPLI between any two electrodes k and m was calculated using the standard formula [42,43]:

(15) $$wPLI_{km}=\frac{\left|E\left[Im\left(S_{km}\right)\right]\right|}{E\left[\left|Im\left(S_{km}\right)\right|\right]}$$

where $wPLI_{km}$ is the weighted Phase Lag Index value between electrode k and electrode m; $S_{km}$ is the cross-spectral complex value between paired channel signals k and m; $Im\left(\cdot \right)$ is the imaginary component of the complex cross-spectrum, reflecting phase lag information between two signals; $E\left[\cdot \right]$ is the ensemble average over all valid time points within the segmented EEG epochs; $\left|\cdot \right|$ is the absolute value operation. The wPLI values range strictly from 0 to 1. A value close to 1 indicates strong and stable phase synchronization and high functional connectivity efficiency between paired brain regions; a value close to 0 represents weak synchronization and independent neural oscillatory activity.

### 2.6. Statistics

RBP analysis and plotting were performed using the default software in the MATLAB Statistical Toolbox. After verifying data normality using the Kolmogorov−Smirnov test, a two-way repeated-measures ANOVA was conducted using SPSS (version 26) to examine the significance of PSD in DOC diagnosis. The effects of frequency band (δ, θ, α) and group (three levels: EMCS, MCS, VS/UWS; two levels: MCS, VS/UWS) on PSD were analyzed, and results were presented as bar charts. If a significant main or interaction effect of group was observed, two-sample *t*-tests were performed as post-hoc analyses with FDR correction. *p*-values that remain significantly different after FDR correction are marked with red asterisks, while those showing no significant difference after correction are marked with blue asterisks. A two-factor mixed-design ANOVA was used to examine the significance of PE, SamEn, wPLI (δ, θ, α), θ-α QPC, and SI in DOC diagnosis. The effects of channel (F3, F4, P3, P4) and group (three or two levels) were analyzed, and results were presented as boxplots. Post-hoc procedures and FDR correction were applied as described above. Paired *t*-tests were performed for the longitudinal comparisons: (1) VS/UWS vs. MCS for PE, SamEn, θ-α QPC, and SI; (2) MCS vs. EMCS for PE and θ-α QPC; two-sample independent *t*-tests were performed for the following comparisons: improved vs. unimproved groups for PE and wPLI.

## 3. Results

### 3.1. Patients Group and Demographics

A total of 153 patients were assigned to the non-improvement group, and 54 were assigned to the improvement group. Notably, the EMCS group was excluded from statistical comparisons involving DOC patients (e.g., Table 1) but was retained in EEG waveform analyses, as their distinct EEG features provide a valuable reference for interpreting neurophysiological patterns across different levels of consciousness. Given that EMCS represents an intermediate state between DOC and wakefulness, we retained the EEG data from 8 EMCS patients to allow for visual comparison with the remaining patients in the non-improvement group, thereby highlighting differences between the VS/UWS and MCS groups. In the unimproved consciousness group, there were 85 patients in the VS/UWS group and 68 patients in the MCS group. In the improved consciousness groups, there were 27 patients in the MCS (recovery from VS/UWS), 11 patients in the EMCS (recovery from VS/UWS), and 16 patients in the EMCS (recovery from MCS). The median (IQR) age of the total number of the groups was 57 (46–66) years and the median (IQR) time from onset to admission was 41 (28–66) days. Detailed etiology distribution for all enrolled participants is provided in Table 1. As shown in Table 1, there were no significant differences ( p > 0.05) between the study groups in terms of gender composition ratio ( $X^{2}$ = 0.07 p = 0.793), age ( H = 4.739 p = 0.449), etiology ( $X^{2}$ = 6.43 p = 0.091), and duration of the disease ( Z = −5.012 p = 0.622). The causes for four of the other etiologies were as follows: pineal tumors, pesticide poisoning, glioma, and viral encephalitis.

### 3.2. Bedside rs-EEG Features in the Classification of DOC

#### 3.2.1. Cross-Sectional Comparison

**Figure 1:**
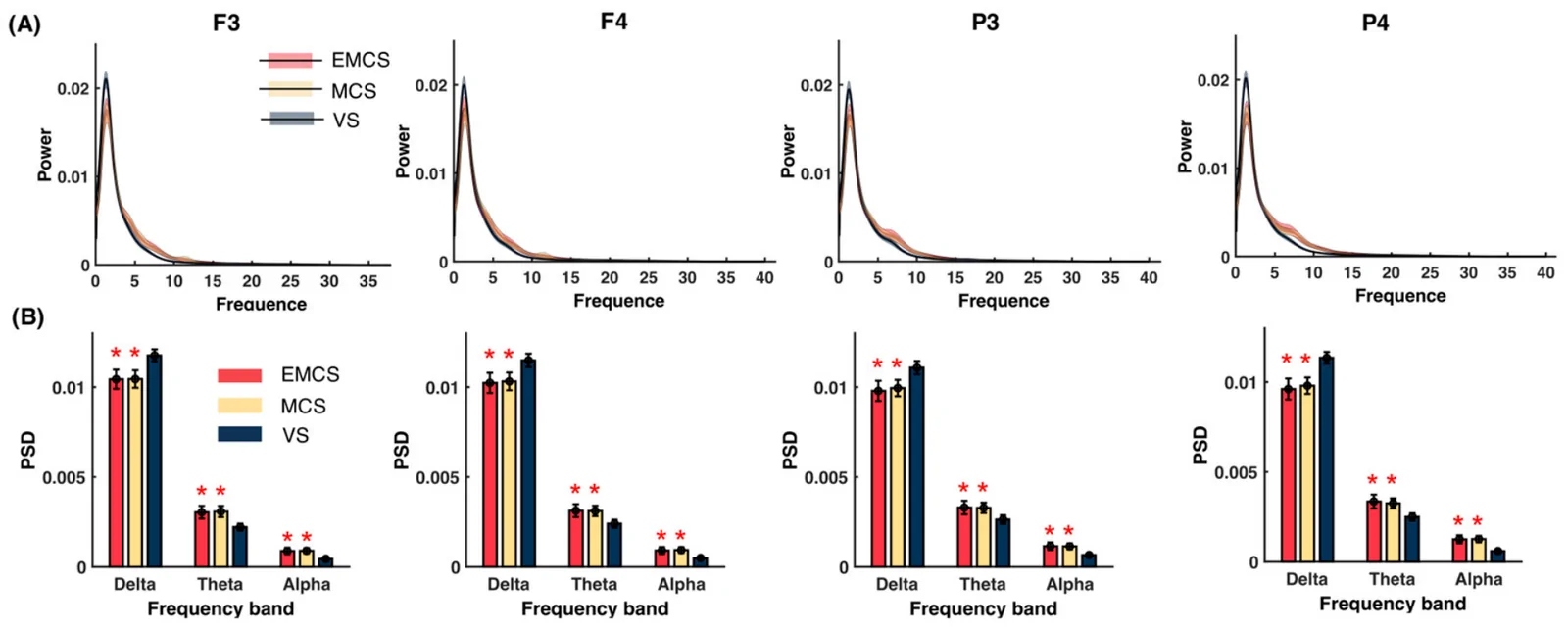
RBP and PSD in the unimproved group and EMCS patients. (**A**) Comparison of RBP between VS/UWS, MCS, and EMCS. (**B**) Comparison of PSD between VS/UWS, MCS, and EMCS. In statistical analyses. The red * indicates that the difference with VS/UWS is still significant after post-hoc tests and FDR correction; *p* < 0.05.

We investigated resting-state EEG features for discriminating consciousness levels via cross-sectional comparisons. VS/UWS patients showed higher RBP of δ waves but lower RBP of θ and α waves compared to MCS and EMCS patients. Additionally, higher-frequency bands exhibited greater amplitudes over parietal versus frontal regions (Figure 1A). A comparison of PSD across leads between groups is shown as follows.

F3 channel: A main effect of frequency band (F [2,158] = 1690.30, p < 0.001, η^2^p = 0.955) and a group-by-band interaction (F [4,316] = 11.24, p < 0.001, η^2^p = 0.125) were observed. Post-hoc tests revealed that EMCS patients exhibited higher θ (0.0030 ± 0.0012 vs. 0.0022 ± 0.0010, p = 0.0002) and α power (0.0009 ± 0.0006 vs. 0.0005 ± 0.0004, p < 0.0001), but lower δ power (0.0104 ± 0.0018 vs. 0.0118 ± 0.0018, *p* = 0.004) than VS/UWS patients. Post-hoc tests revealed that compared with VS/UWS patients, MCS patients showed lower δ power (0.0105 ± 0.0023 vs. 0.0118 ± 0.0018, p = 0.0001), higher θ power (0.0030 ± 0.0015 vs. 0.0022 ± 0.0010, p < 0.0001), and higher α power (0.0009 ± 0.0007 vs. 0.0005 ± 0.0004, p < 0.0001). No significant differences emerged between EMCS and MCS.

F4 channel: The main effect of frequency band (F [2,158] = 1488.10, p < 0.001, η^2^p = 0.949) and group-by-band interaction (F [4,316] = 8.21, p < 0.001, η^2^p = 0.094) were significant. Post-hoc tests showed that EMCS patients had lower δ (0.0102 ± 0.0019 vs. 0.0115 ± 0.0020, p = 0.0017), and higher θ (0.0031 ± 0.0012 vs. 0.0024 ± 0.0012, p = 0.0028) and α power (0.0009 ± 0.0007 vs. 0.0005 ± 0.0004, p < 0.0001) than VS/UWS. Post-hoc tests showed that MCS patients, relative to VS/UWS patients, demonstrated lower δ power (0.0103 ± 0.0023 vs. 0.0115 ± 0.0020, p = 0.001), higher θ power (0.0031 ± 0.0014 vs. 0.0024 ± 0.0012, p < 0.0001), and higher α power (0.0009 ± 0.0008 vs. 0.0005 ± 0.0004, p < 0.0001). No EMCS-MCS differences were found.

P3 channel: A main effect of frequency band (F [2,158] = 1307.10, p < 0.001, η^2^p = 0.943) and group-by-band interaction (F [4,316] = 8.18, p < 0.001, η^2^p = 0.093) were observed. Post-hoc tests revealed that EMCS patients displayed lower δ (0.0098 ± 0.0019 vs. 0.0111 ± 0.0020, p = 0.0013), and higher θ (0.0033 ± 0.0013 vs. 0.0026 ± 0.0013, p = 0.0099) and α power (0.0012 ± 0.0007 vs. 0.0007 ± 0.0005, p < 0.0001) than VS/UWS. Post-hoc tests revealed that MCS patients exhibited lower δ power (0.0100 ± 0.0022 vs. 0.0111 ± 0.0020, p = 0.001), higher θ power (0.0033 ± 0.0014 vs. 0.0026 ± 0.0013, p < 0.0001), and higher α power (0.0011 ± 0.0008 vs. 0.0007 ± 0.0005, p < 0.0001) compared with VS/UWS patients. No significant differences were observed between EMCS and MCS.

P4 channel: The main effect of frequency band (F [2,158] = 1402.60, p < 0.001, η^2^p = 0.947) and group-by-band interaction (F [4,316] = 15.63, p < 0.001, η^2^p = 0.165) were significant. Post-hoc tests revealed that EMCS patients exhibited lower δ (0.0096 ± 0.0020 vs. 0.0114 ± 0.0017, p < 0.0001), and higher θ (0.0034 ± 0.0013 vs. 0.0025 ± 0.0011, p = 0.0003) and α power (0.0012 ± 0.0008 vs. 0.0006 ± 0.0005, p < 0.0001) than VS/UWS. Post-hoc tests revealed that MCS patients showed lower δ power (0.0098 ± 0.0022 vs. 0.0114 ± 0.0017, p < 0.0001), higher θ power (0.0033 ± 0.0013 vs. 0.0025 ± 0.0011, p = 0.0001), and higher α power (0.0013 ± 0.0010 vs. 0.0006 ± 0.0005, p < 0.0001) compared to VS/UWS patients (Figure 1B). No significant differences were observed between EMCS and MCS patients across all channels (Figure 1B).

**Figure 2:**
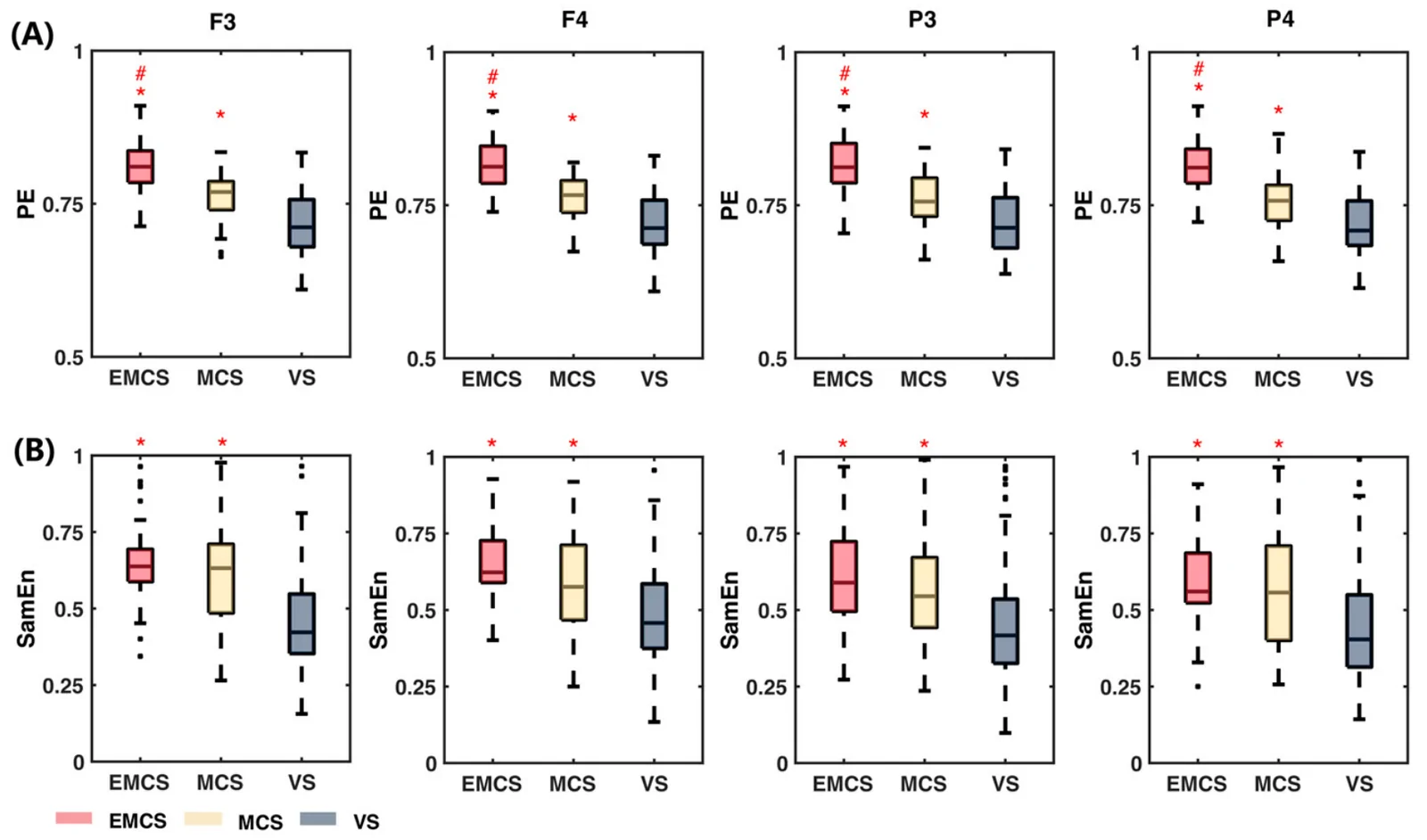
PE and SamEn in the unimproved group and EMCS patients. (**A**) Comparison of PE between VS/UWS, MCS, and EMCS. (**B**) Comparison of SamEn between VS/UWS, MCS, and EMCS. In the statistical analysis, the red * indicates a significant difference in the results compared to VS/UWS, while still being *p* < 0.05 after post-hoc tests and FDR correction. # indicates a significant difference in the statistical analysis compared to MCS, while still being *p* < 0.05 after post-hoc tests and FDR correction.

Notably, significant differences in PE values across all leads were observed among the three groups of DOC patients with distinct levels of consciousness. A main effect of group was found (F [2,158] = 6.14, p < 0.001, η^2^p = 0.040), indicating that PE values significantly differed among the three consciousness levels. However, no significant interaction effect was observed between group and channel (F [6,474] = 1.08, p = 0.395, η^2^p = 0.022). Post-hoc *t*-tests within each channel revealed that the EMCS group exhibited significantly higher PE values than the VS/UWS group across all leads (F3: 0.8012 ± 0.0042 vs. 0.7015 ± 0.0068; F4: 0.8023 ± 0.0033 vs. 0.7014 ± 0.0065; P3: 0.8020 ± 0.0059 vs. 0.7015 ± 0.0058; P4: 0.7995 ± 0.0037 vs. 0.6999 ± 0.0060; all p < 0.0001). Similarly, PE values in the EMCS group were significantly higher than those in the MCS group for all leads (F3: 0.8012 ± 0.0042 vs. 0.7655 ± 0.0028, p = 0.0008; F4: 0.8023 ± 0.0033 vs. 0.7588 ± 0.0026, p = 0.0005; P3: 0.8020 ± 0.0059 vs. 0.7505 ± 0.0037, p = 0.0002; P4: 0.7995 ± 0.0037 vs. 0.7521 ± 0.0047, p = 0.005) (Figure 2A). Simultaneously, the MCS group exhibited significantly higher PE values across all leads compared to the VS/UWS group ( P (F3) = 0.0012; P (F4) = 0.0018; P (P3) = 0.0023; P (P4) = 0.0023) (Figure 2A). This difference remained statistically significant after post-hoc testing and FDR correction.

SamEn analysis revealed a significant main effect of consciousness level (F [2,158] = 16.25, p < 0.001, η^2^p = 0.211). However, no significant interaction was found between consciousness level and channel (F [6,474] = 1.29, p = 0.121, η^2^p = 0.032). Post-hoc comparisons were conducted 12 times, with 8 FDR-corrected comparisons, as shown in Figure 2B. Post-hoc *t*-tests within each channel revealed that the EMCS group exhibited significantly higher SamEn values than the VS/UWS group across all leads (F3: 0.6325 ± 0.0013 vs. 0.4012 ± 0.0023; F4: 0.6300 ± 0.0016 vs. 0.4025 ± 0.0035; P3: 0.5820 ± 0.0035 vs. 0.3890 ± 0.0038; P4: 0.5510 ± 0.0015 vs. 0.3840 ± 0.0030; all p < 0.0001). Similarly, SamEn values in the MCS group were significantly higher than those in the VS/UWS group for all leads (F3: 0.5395 ± 0.0038 vs. 0.4012 ± 0.0023, p < 0.0001; F4: 0.5720 ± 0.0028 vs. 0.4025 ± 0.0035, p = 0.0002; P3: 0.5395 ± 0.0038 vs. 0.3890 ± 0.0038, p = 0.0002; P4: 0.5460 ± 0.0028 vs. 0.3840 ± 0.0030, p < 0.0001). However, no significant differences in SamEn values were observed between the EMCS and MCS groups.

**Figure 3:**
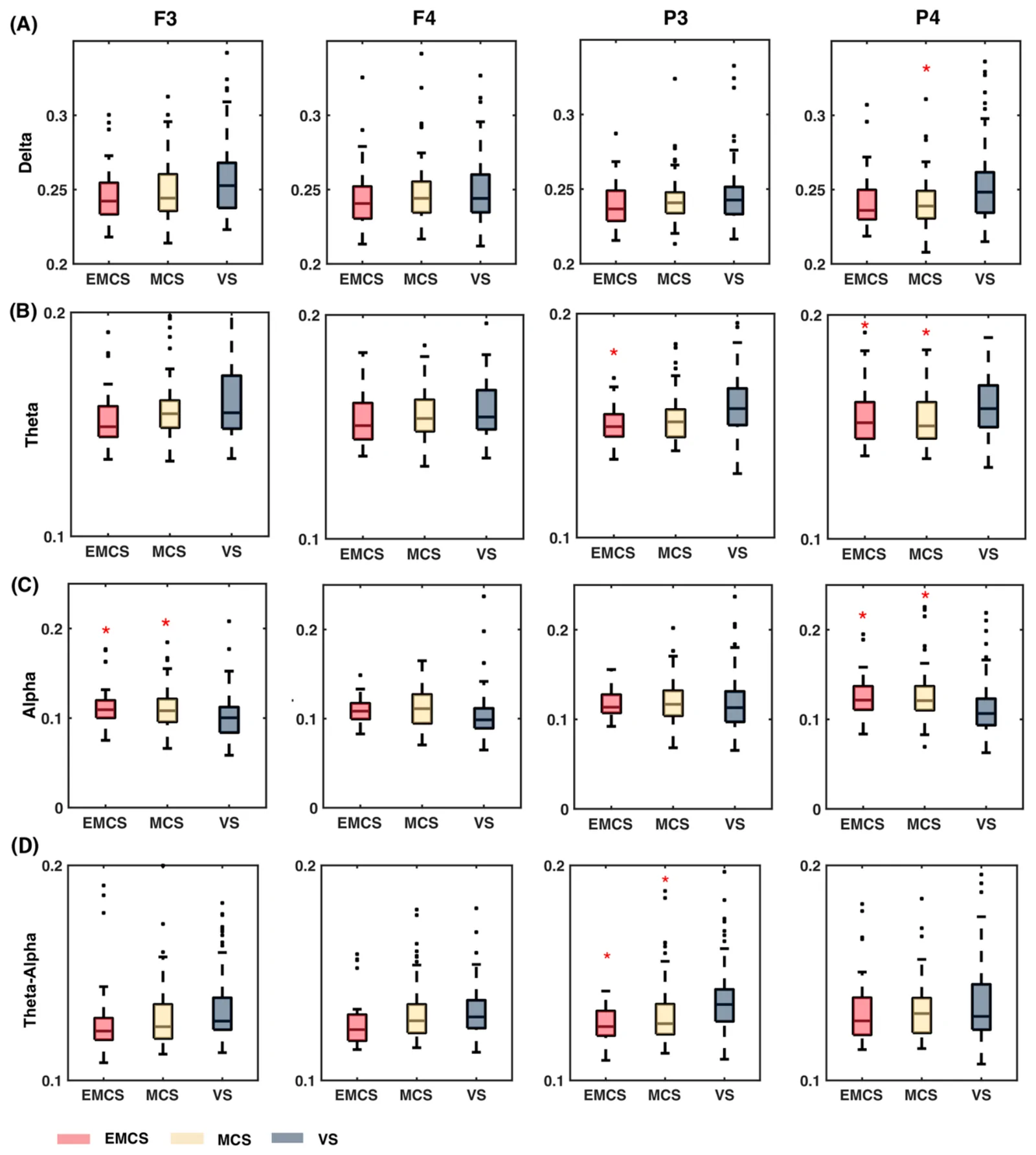
QPC/QPSC in the unimproved group and EMCS patients. (**A**) Comparison of δ-QPSC between VS/UWS, MCS, and EMCS. (**B**) Comparison of θ-QPSC between VS/UWS, MCS, and EMCS. (**C**) Comparison of α-QPSC between VS/UWS, MCS, and EMCS. (**D**) Comparison of QPC between VS/UWS and QPC of θ-α between MCS and EMCS. The red * indicates a significant difference in the statistical analysis compared to VS/UWS while still *p* < 0.05 after post-hoc tests and FDR correction.

In the δ-QPSC analysis results, the within-subject effect across channels was F [3,474] = 6.85, p < 0.001, η^2^p = 0.063; the between-subjects effect across consciousness levels was F [2,158] = 2.14, p = 0.123, η^2^p = 0.040; the interaction effect between consciousness level groups and channel was F [6,474] = 0.90, p = 0.495, η^2^p = 0.017. Post-hoc comparisons were conducted 12 times with 1 FDR-corrected comparison, as shown in Figure 3A. Post-hoc *t*-tests within each group showed that the δ-QPSC at MCS (P4: mean ± 1 SD: 0.2423 ± 0.0020) was significantly lower only at the P4 position compared to VS/UWS (P4: mean ± 1 SD: 0.2547 ± 0.0036) ( p = 0.0327). In the θ-QPSC analysis results, the within-subject effects across channels were not significant (F [3,474] = 1.95, p = 0.122, η^2^p = 0.019), while a significant between-subjects main effect for consciousness level was observed (F [2,158] = 5.55, p = 0.005, η^2^p = 0.098). The interaction between consciousness level and channel was not significant (F [6,474] = 0.32, p = 0.928, η^2^p = 0.006). Post-hoc comparisons were conducted 12 times, with 2 FDR-corrected comparisons, as shown in Figure 3B. Post-hoc *t*-tests revealed that the EMCS group exhibited significantly lower θ-QPSC values than the VS/UWS group at the P3 (0.1502 ± 0.0014 vs. 0.1616 ± 0.0021, p = 0.0097) and P4 leads (0.1539 ± 0.0021 vs. 0.1648 ± 0.0029, p = 0.0245), while the MCS group showed significantly lower values than the VS/UWS group only at the P4 lead (0.1555 ± 0.0024 vs. 0.1648 ± 0.0028, p = 0.0199). In the α-QPSC analysis results, a significant within-subject effect across channels was observed (F [3,474] = 13.87, p < 0.001, η^2^p = 0.120), while the interaction between consciousness level and channel approached but did not reach significance (F [6,474] = 2.10, p = 0.053, η^2^p = 0.040). Post-hoc comparisons were conducted 12 times, with 4 FDR-corrected comparisons, as shown in Figure 3C. Post-hoc *t*-tests revealed that both the EMCS and MCS groups exhibited significantly higher α-QPSC values than the VS/UWS group at the F3 and P4 leads. Specifically, differences between EMCS and VS/UWS groups were observed at F3 (0.1141 ± 0.0045 vs. 0.1012 ± 0.0026, p = 0.0111) and P4 (0.1293 ± 0.0059 vs. 0.1110 ± 0.0031, p = 0.0037); differences between MCS and VS/UWS groups were observed at F3 (0.1138 ± 0.0050 vs. 0.1012 ± 0.0026, p = 0.0195) and P4 (0.1270 ± 0.0039 vs. 0.1110 ± 0.0031, p = 0.0013). In the θ-α QPC analysis results, a significant within-subject effect across channels was observed (F [3,474] = 2.82, p = 0.039, η^2^p = 0.027), while the between-subjects main effect for consciousness level was not significant (F [2,158] = 2.97, p = 0.056, η^2^p = 0.055). The interaction between consciousness level and channel was not significant (F [6,474] = 0.95, p = 0.460, η^2^p = 0.018). Post-hoc comparisons were conducted 12 times, with 2 FDR-corrected comparisons, as shown in Figure 3D. Post-hoc *t*-tests revealed that both the EMCS (0.1266 ± 0.0015, p = 0.0007) and MCS groups (0.1316 ± 0.0020, p = 0.0117) exhibited significantly lower θ-α QPC values than the VS/UWS group (0.1396 ± 0.0023) at the P3 lead.

**Figure 4:**
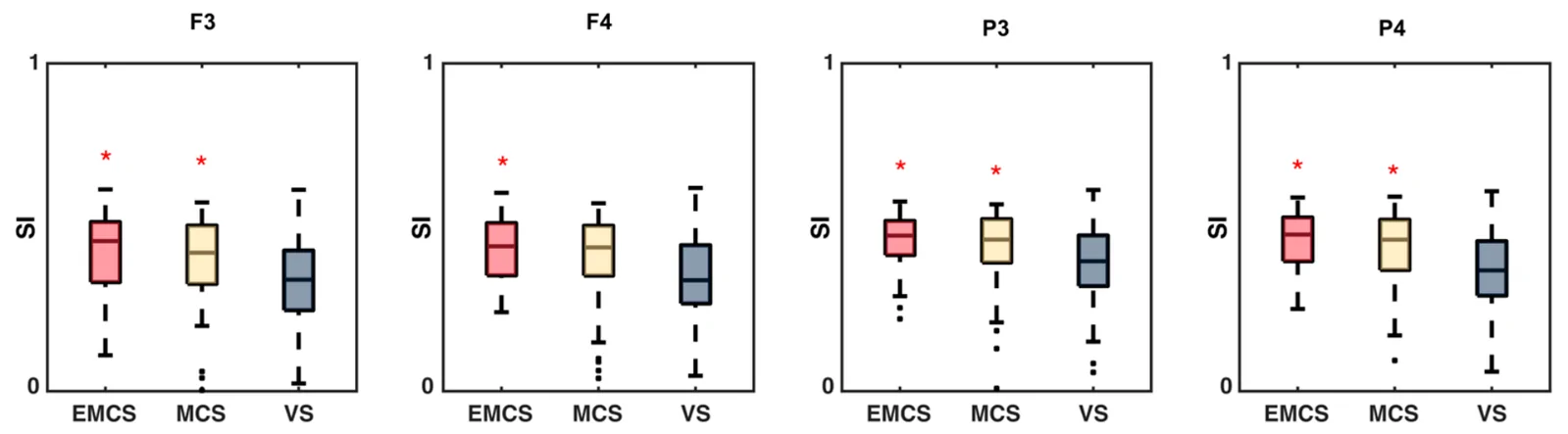
SI in the unimproved group and EMCS patients. The red * indicates a significant difference in the statistical analysis compared to VS/UWS while still *p* < 0.05 after post-hoc tests and FDR correction.

In the SI analysis results, a significant within-subject effect across channels was observed (F [3,474] = 9.22, p < 0.001, η^2^p = 0.083), as well as a significant between-subjects main effect for consciousness level (F [2,158] = 4.61, p = 0.012, η^2^p = 0.073). The interaction between consciousness level and channel was not significant (F [6,474] = 1.03, p = 0.408, η^2^p = 0.020). Post-hoc comparisons were conducted 12 times, with 7 FDR-corrected comparisons, as shown in Figure 4. Post-hoc *t*-tests revealed that the EMCS group exhibited significantly higher SI values than the VS/UWS group across all leads: F3 (0.5676 ± 0.0086 vs. 0.5368 ± 0.0057, p = 0.0037), F4 (0.5722 ± 0.0071 vs. 0.5413 ± 0.0059, p = 0.0035), P3 (0.5822 ± 0.0062 vs. 0.5541 ± 0.0056, p = 0.0039), and P4 (0.5862 ± 0.0056 vs. 0.5486 ± 0.0051, p = 0.0003). The MCS group showed significantly higher SI values than the VS/UWS group at the F3 (0.5606 ± 0.0063 vs. 0.5368 ± 0.0057, p = 0.0056), P3 (0.5760 ± 0.0056 vs. 0.5541 ± 0.0056, p = 0.0069), and P4 leads (0.5760 ± 0.0053 vs. 0.5486 ± 0.0051, p = 0.0004).

**Figure 5:**
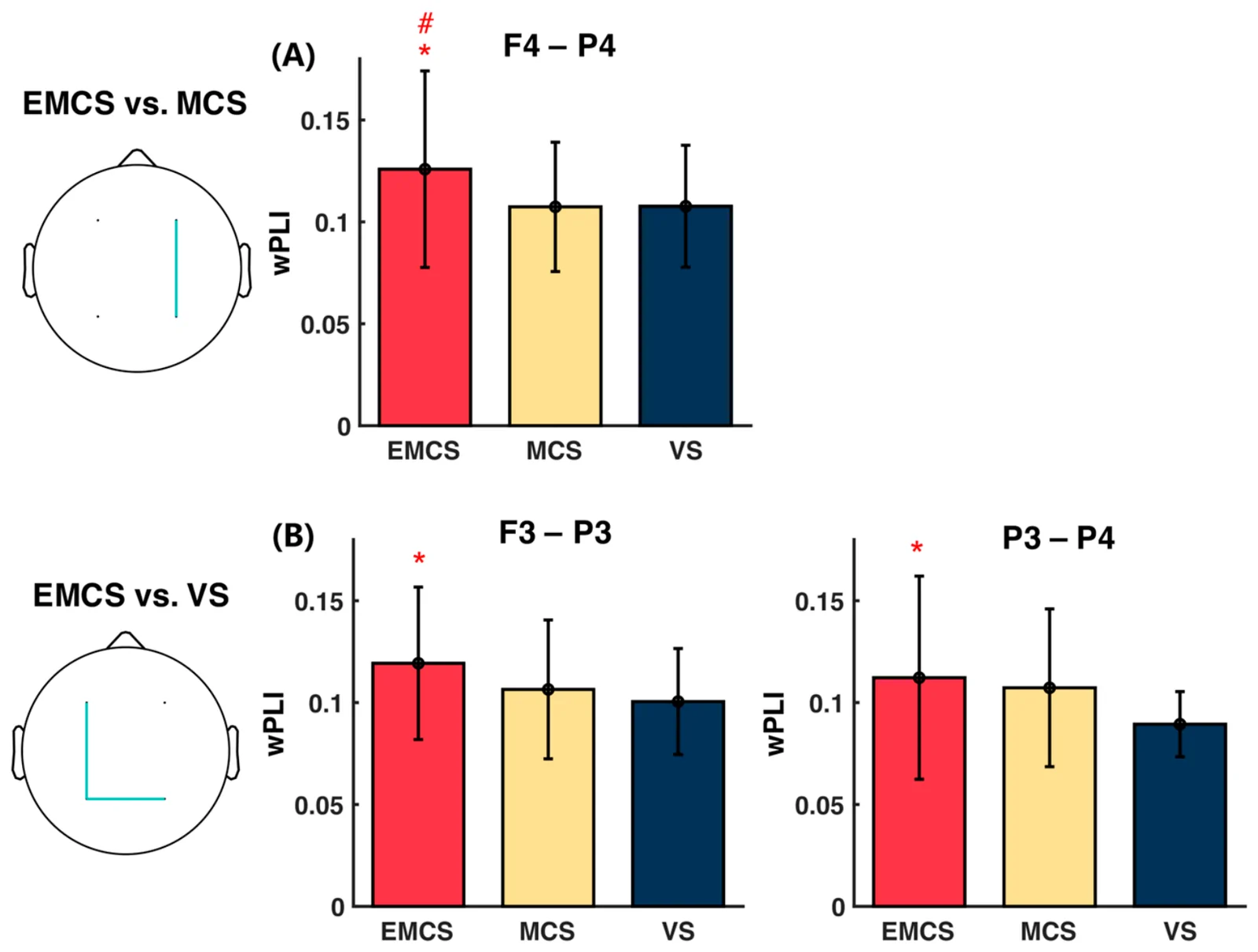
wPLI in the unimproved group and EMCS patients. (**A**) Comparison of wPLI between MCS and EMCS. (**B**) Comparison of wPLI between VS/UWS and EMCS. In the statistical analysis, the red * indicates a significant difference in the results compared to VS/UWS, while still being *p* < 0.05 after post-hoc tests and FDR correction. # indicates a significant difference in the statistical analysis compared to MCS, while still being *p* < 0.05 after post-hoc tests and FDR correction.

In the wPLI analysis results, a significant within-subject effect across channel pairs was observed (F [3,474] = 8.66, p < 0.001, η^2^p = 0.062), along with a significant between-subjects main effect for consciousness level (F [2,158] = 3.88, p = 0.026, η^2^p = 0.042). A significant interaction was found between consciousness level and channel pairs (F [6,474] = 2.64, p = 0.033, η^2^p = 0.039). Post-hoc comparisons were conducted 12 times, with 3 FDR-corrected comparisons, as shown in Figure 5. Post-hoc *t*-tests revealed that the EMCS group exhibited significantly higher wPLI values than both the MCS and VS/UWS groups at the F4−P4 lead (EMCS: 0.1259 ± 0.0482 vs. MCS: 0.1074 ± 0.0317, p = 0.0104; vs. VS/UWS: 0.1077 ± 0.0299, p = 0.0093) (Figure 5A). Additionally, the EMCS group showed significantly higher wPLI values than the VS/UWS group at the F3−P3 (0.1193 ± 0.0374 vs. 0.1005 ± 0.0260, p = 0.0077) and P3−P4 leads (0.1122 ± 0.0498 vs. 0.0894 ± 0.0160, p = 0.0072) (Figure 5B). No significant differences were observed in the remaining electrode channel pairs across other groups.

#### 3.2.2. Longitudinal Comparison

**Figure 6:**
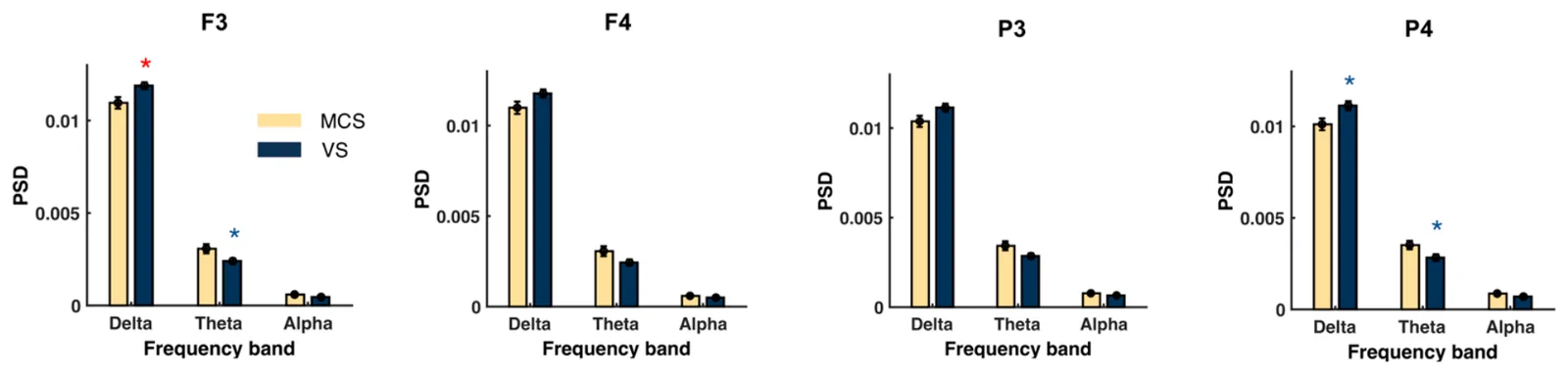
Longitudinal comparison of PSD between VS/UWS and MCS states. The red * indicates significant differences remaining after FDR correction. The blue * indicates no significant differences remaining after post-hoc tests and FDR correction.

In order to mitigate the effect of interindividual EEG differences, the present study focused on the longitudinal comparison of bedside rs-EEG characteristics of the same patient in different conscious states. First, bedside rs-EEG data from the same patient under different states of consciousness were compared. Delta wave PSD at F3 and P4 leads during VS/UWS (F3: mean ± 1 SD: 0.0119 ±0.0011; P4: 0.0111 ± 0.0015) was significantly higher than during MCS (F3: 0.0110 ± 0.0018; P4: 0.0101 ± 0.0019) ( P (F3) = 0.0097; P (P4) = 0.0125). Conversely, θ wave PSD in VS/UWS status at F3 and P4 leads (F3: 0.0024 ± 0.0009; P4: 0.0028 ± 0.0011) was significantly lower than that in the MCS state (F3: 0.0031 ± 0.0015; P4: 0.0035 ± 0.0014) ( P (F3) = 0.0217; P (P4) = 0.0234). However, after post-hoc testing and FDR correction, only the PSD of δ waves in channel F3 remained significantly different (12 post-hoc tests, 4 FDR-corrected tests), as shown in Figure 6.

**Figure 7:**
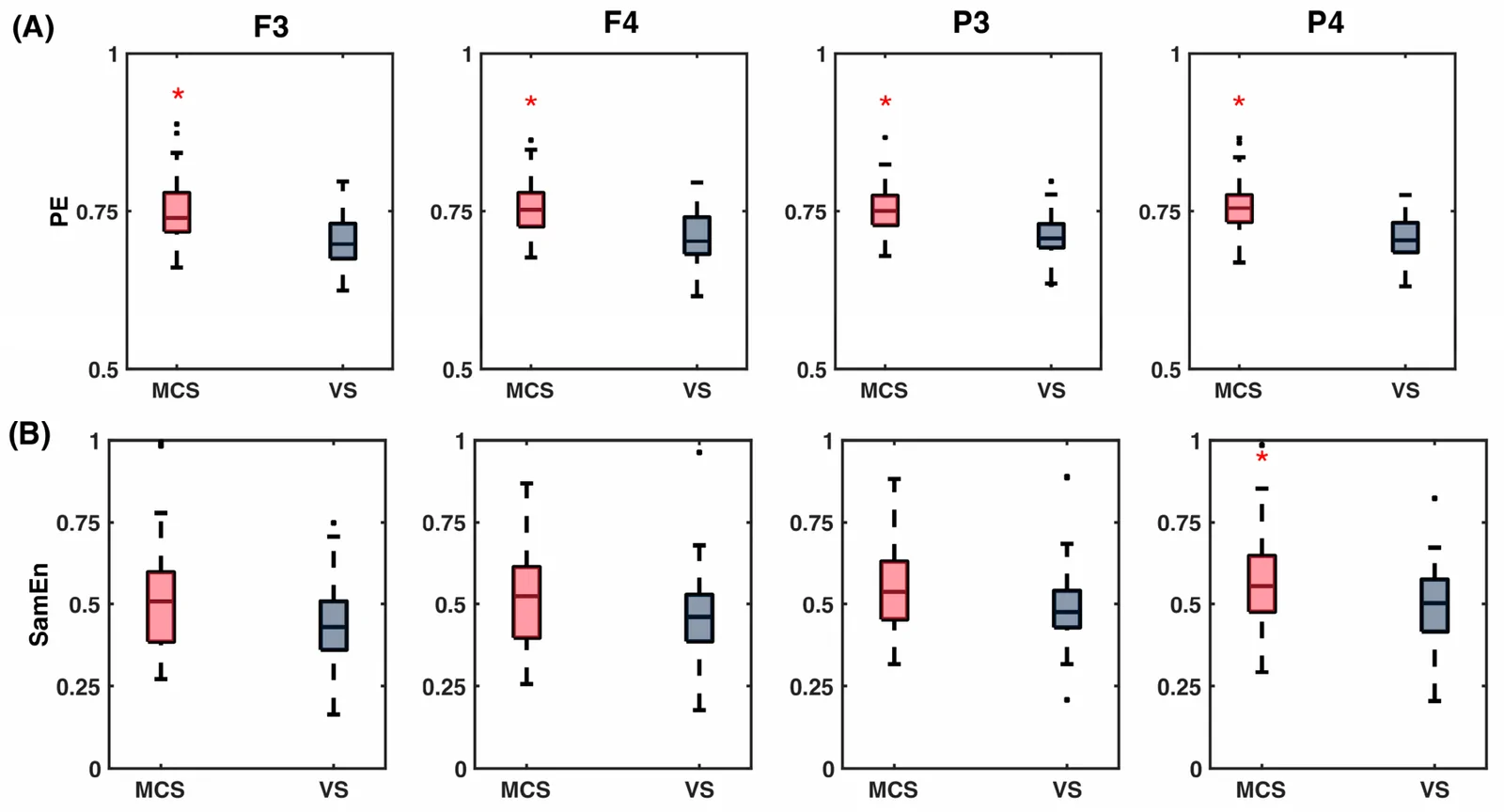
Longitudinal comparison of PE and SamEn between the VS/UWS state and the MCS state. (**A**) Comparison of PE between VS/UWS and MCS states. (**B**) Comparison of SamEn between VS/UWS and MCS states. Red * marks indicate significant differences compared to the VS/UWS group in statistical analysis, with *p* < 0.05 after post-hoc tests and FDR correction.

**Figure 8:**
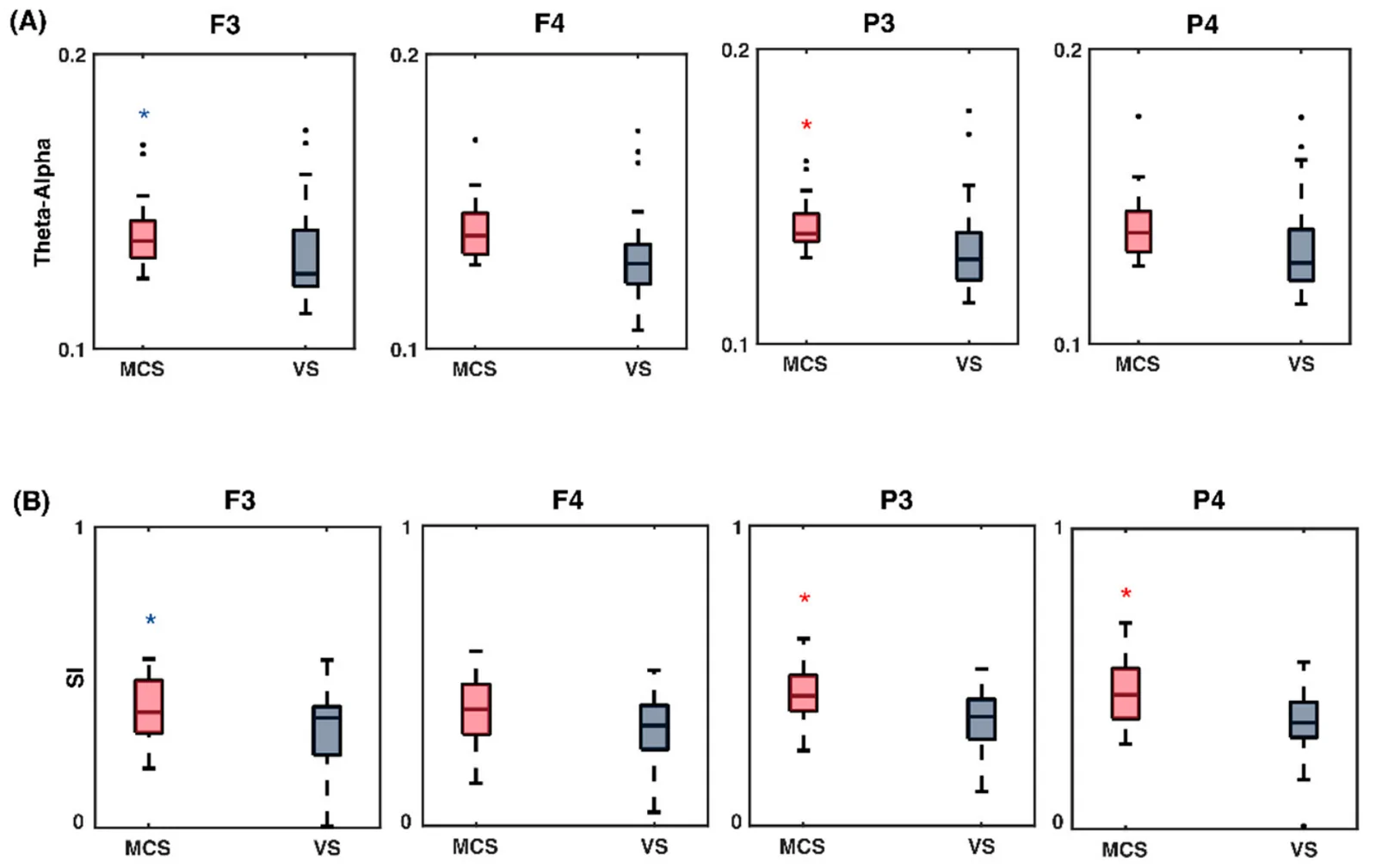
Longitudinal comparison of QPC and SI between VS/UWS and MCS states. (**A**) Comparison of θ-α band QPC values between VS/UWS and MCS states. (**B**) Comparison of SI values between VS/UWS and MCS states. The red * indicates significant differences compared to the VS/UWS group in statistical analysis, remaining significant after post-hoc tests and FDR correction. The blue * indicates significant differences compared to the VS/UWS group in statistical analysis, remaining significant after post-hoc tests and FDR correction.

When patients were in the MCS state, the PE values for all leads (F3: 0.7480 ± 0.0012; F4: 0.7510 ± 0.0011; P3: 0.7500 ± 0.0010; P4: 0.7525 ± 0.0015) were significantly higher than those in the VS/UWS state (F3: 0.7120 ± 0.0013; F4: 0.7100 ± 0.0013; P3: 0.7100 ± 0.0009; P4: 0.7088 ± 0.0010; ( P (F3) = 0.0035; P (F4) = 0.0012; P (P3) = 0.0013; P (P4) = 0.0009). Post-hoc comparisons were conducted four times, with FDR correction applied four times, as shown in Figure 7A. The SamEn value at P4 in the MCS state (P4: 0.5500 ± 0.0028) was significantly higher than that in the VS/UWS state (P4: 0.5012 ± 0.0019) ( p = 0.0008). No significant differences were observed in the remaining leads, as shown in Figure 7B. Post-hoc comparisons were performed four times, with one FDR-corrected comparison. MCS patients exhibited significantly higher θ-α wave QPC values in the F3 and P3 leads (F3: 0.1410 ± 0.0012; P3: 0.1438 ± 0.0010) compared to VS/UWS states (F3: 0.1255 ± 0.0020; P3: 0.1295 ± 0.0018) ( P (F3) = 0.0008; P (P3) = 0.0010). However, the difference in the F3 lead did not remain significant after FDR correction (corrected p > 0.05), as shown in Figure 8A. The number of post-hoc comparisons was 4, and the number of FDR corrections was 2. In the MCS state, the SI values at leads F3, P3, and P4 (F3: 0.5600 ± 0.0055; P3: 0.5675 ± 0.0052; P4: 0.5680 ± 0.0055) were significantly higher than those in the VS/UWS state (F3: 0.5410 ± 0.0057; P3: 0.5408 ± 0.0045; P4: 0.5400 ± 0.0042) ( P (F3) = 0.0122; P (P3) = 0.0065; P (P4) = 0.0032). However, after FDR correction, the difference in F3 lead was deemed statistically insignificant ( p > 0.05), as shown in Figure 8B. The number of post-hoc comparisons was 4, and the number of FDR-corrected comparisons was 3.

**Figure 9:**
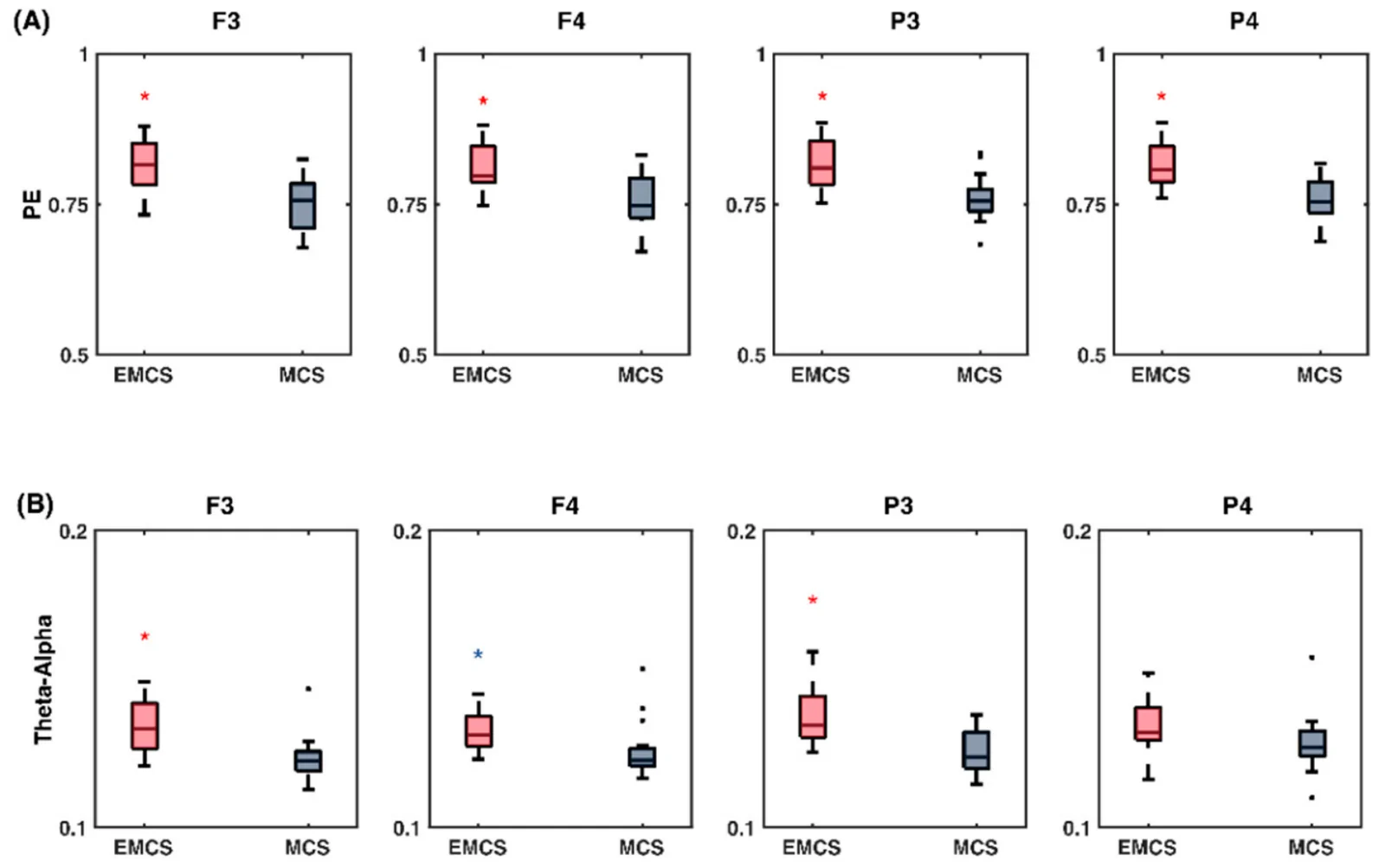
Longitudinal comparison of PE and QPC between MCS and EMCS states. (**A**) Comparison of PE values between MCS and EMCS states. (**B**) Comparison of QPC values in the θ-α band between MCS and EMCS states. The red * indicates significant differences compared to MCS that remain significant after post-hoc tests and FDR correction. The blue * indicates significant differences compared to MCS that do not remain significant after post-hoc tests and FDR correction.

All lead PE values under EMCS status (F3: 0.8025 ± 0.0036; F4: 0.7985 ± 0.0028; P3: 0.8011 ± 0.0022; P4: 0.8011 ± 0.0020) were significantly higher than those in the MCS state (F3: 0.7565 ± 0.0035; F4: 0.7488 ± 0.0036; P3: 0.7560 ± 0.0012; P4: 0.7562 ± 0.0015) ( P (F3) = 0.0003; P (F4) = 0.0014; P (P3) = 0.0004; P (P4) = 0.0004), as shown in Figure 9A. Post-hoc comparisons were conducted four times, with FDR correction applied four times.

QPC values of θ-α in F3, F4, and P3 leads during EMCS (F3: 0.1357 ± 0.0015; F4: 0.1320 ± 0.0012; P3: 0.1380 ± 0.0016) were significantly higher than those in the MCS state (F3: 0.1268 ± 0.0006; F4: 0.1268 ± 0.0003; P3: 0.1268 ± 0.0010) ( P (F3) = 0.0008; P (F4) = 0.0021; P (P3) = 0.0007). However, the difference in F4 lead was not statistically significant after FDR correction, as shown in Figure 9B. The number of post-hoc comparisons was 4, and the number of FDR-corrected comparisons was 3.

### 3.3. Bedside rs-EEG Features in Prognosis of DOC

**Figure 10:**
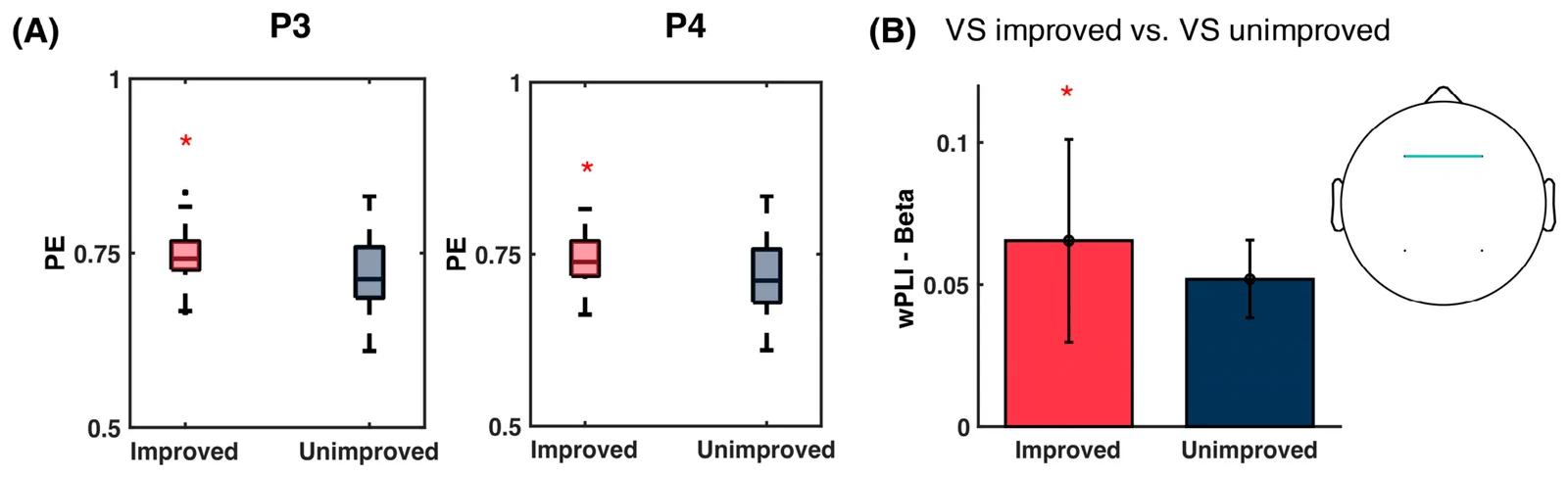
rs-EEG before and after improvement in patients with and without consciousness at the same level of consciousness. (**A**) Comparison of PE between VS/UWS (unimproved) and VS/UWS (improved) states. (**B**) Comparison of wPLI-β between VS/UWS (unimproved) and VS/UWS (improved) states. Red * marks indicate significant differences between the two groups that remain significant after post-hoc tests and FDR correction.

To further explore the bedside rs-EEG characteristics associated with DOC prognosis, we performed a comparison of the EEG characteristics of VS/UWS patients and MCS patients whose consciousness did not improve during hospitalization (i.e., VS/UWS (unimproved) and MCS (unimproved)) with those who showed an improvement in their consciousness during hospitalization (i.e., VS/UWS (improved) and MCS (improved)). Results revealed that the PE values in the VS/UWS (unimproved) group at leads P3 and P4 (P3: mean ± 1 SD: 0.7010 ± 0.0055; P4: 0.7005 ± 0.0056) were significantly lower than those in the VS/UWS (improved) group (P3: 0.7475 ± 0.0036; P4: 0.7475 ± 0.0032) ( P (P3) = 0.0033; P (P4) = 0.0030), as shown in Figure 10A. The β-wPLI values in the F3−F4 lead for the VS/UWS (unimproved) group (F3−F4: 0.0519 ± 0.0136) were significantly lower than those in the VS/UWS (improved) group (F3−F4: 0.0653 ± 0.0358) ( p = 0.0031), as shown in Figure 10B.

**Figure 11:**
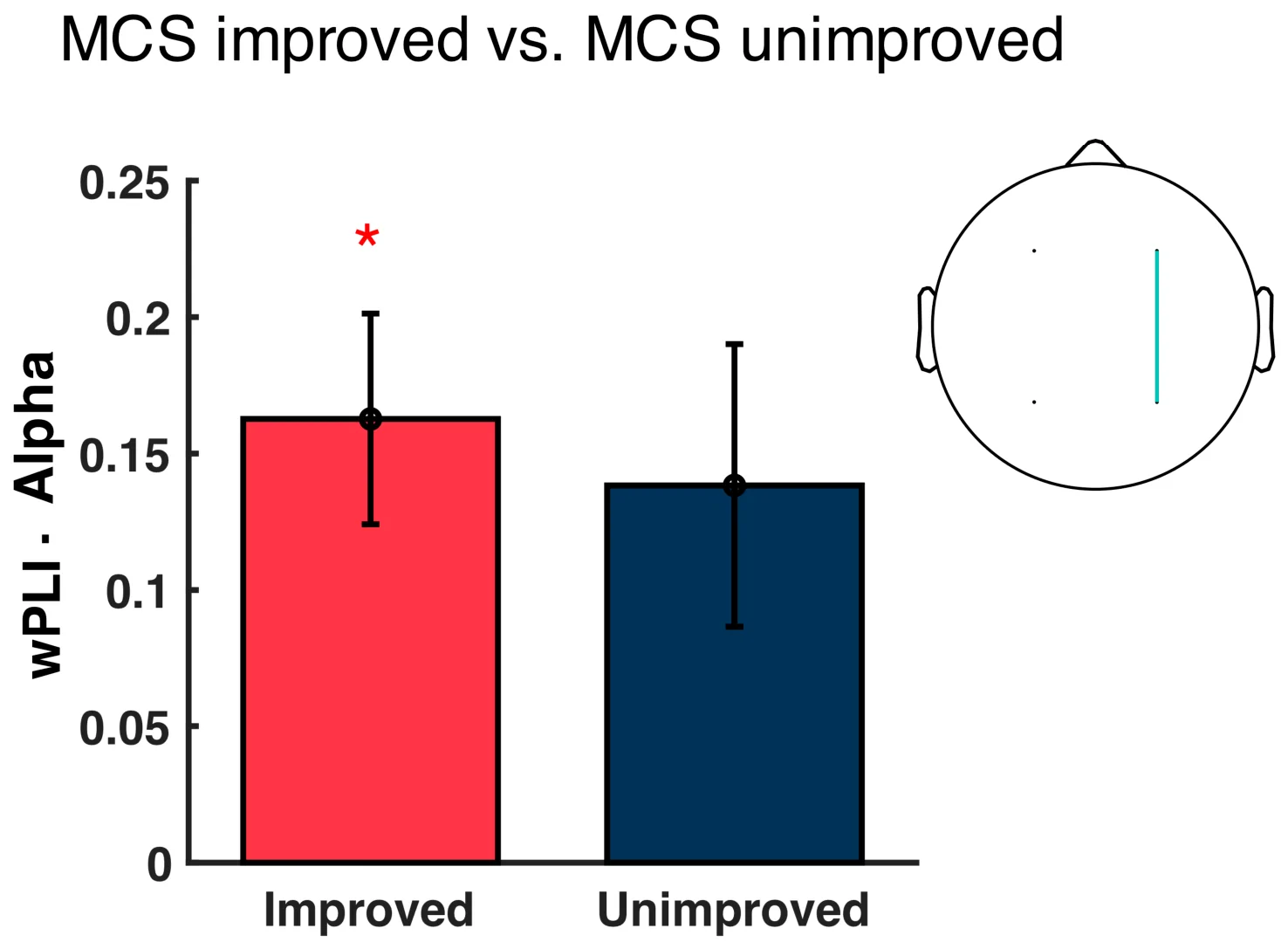
Comparison of wPLI-α between MCS (unimproved) and MCS (improved). Red * marks indicate significant differences between the two groups that remain significant after post-hoc tests and FDR correction.

The MCS (improved) group exhibited significantly higher α-wPLI values in the F4−P4 lead (F4−P4: 0.1625 ± 0.0322) than patients in the MCS (unimproved) group (F4−P4: 0.1412 ± 0.0413) ( p = 0.0021), as shown in Figure 11. No significant differences were observed between patients in the improved group and the unimproved group in other indicators.

## 4. Discussion

The findings derived from our analysis yielded multiple rs-EEG features that contribute to the diagnosis of DOC and the forecasting of prognosis in consciousness. Firstly, concerning the diagnostic assessment of consciousness levels, the RBP of the δ wave was found to be higher in patients with VS/UWS compared to those in MCS and EMCS. Moreover, the RBP of θ and α waves exhibited a decrease in patients with VS/UWS in comparison to those with MCS and EMCS. This trend was observed both in cross-sectional analyses involving distinct individuals and in longitudinal assessments of varying levels of consciousness within the same individual. The results align with prior research conducted by other scholars [23]. In our study, only δ, θ, and α waves were detected, with the absence or insignificance of higher frequency β and γ waves. This observation may be attributed to the typically diminished level of consciousness in patients with DOC, wherein β and γ wave activity tends to increase solely during wakefulness or rapid eye movement sleep [44]. Simultaneously, it was observed that the amplitudes of θ and α waves in the parietal lobe exceeded those in the frontal lobe. These results align with prior research conducted by other investigators [45]. The finding could be associated with cortical excitation, as neural oscillatory activity in the θ and α bands has been linked to executive function [46,47]. Some studies have proposed a connection between α band activity in the parietal lobe and attentional orienting and cognitive control processes, indicating a potential relationship between parietal lobe activity and cortical excitation [47,48]. However, despite these differences, the diagnostic efficacy of RBP remains limited.

The present cross-sectional and longitudinal comparisons reveal a notable elevation in PSD of δ wave among patients in VS/UWS in comparison to individuals exhibiting higher levels of consciousness. Furthermore, a notable reduction in PSD of θ and α wave was evident in individuals with VS/UWS in comparison to those exhibiting heightened levels of consciousness. This finding aligns with the outcomes reported by prior investigators [24]. The heightened level of consciousness observed in patients with DOC is believed to be associated with an elevation in parieto-occipital PSD [49]. The outcomes of PSD closely resemble those of RBP, but PSD allows for quantitative evaluation to indicate alterations in consciousness levels. The study results revealed that while PSD could effectively discern individuals in VS/UWS, it lacked the ability to differentiate between patients in MCS and EMCS. Hence, the diagnostic value of PSD in DOC is limited.

The PE is a nonlinear metric derived from symbolic dynamics that quantifies the complexity of a time series by evaluating the regularity or randomness of ordinal patterns among consecutive data points. Neurophysiologically, PE reflects the diversity and predictability of neuronal ensemble firing patterns. During heightened consciousness, balanced brain network dynamics generate rich spatiotemporal activity patterns, yielding higher PE values. This study demonstrates that PE serves as a crucial objective bedside rs-EEG indicator for distinguishing different levels of consciousness in patients with impaired consciousness, applicable both across individuals and within the same individual. Furthermore, there was a positive correlation between the PE value and the patient’s level of consciousness, indicating that higher levels of consciousness were associated with higher PE values. This relationship may be attributed to the increasing complexity of the patient’s EEG as their level of consciousness rises [39,40]. At the same time, PE served as a valid indicator of different levels of consciousness in both the frontal and parietal lobes. This is consistent with previous findings involving schizophrenic patients, where the greatest differences in PE complexity were observed in the frontal and occipital regions, while the smallest differences were observed in the central region [50]. In conclusion, this study concluded that PE can serve as an objective bedside EEG indicator for diagnosing the level of consciousness in patients with DOC. To assess the prognostic value of bedside rs-EEG characteristics, we compared these features between patients who regained consciousness and those who did not. The study revealed that patients in VS/UWS who exhibited improvements in consciousness had notably higher PE at parietal leads. This suggests that the PE in the parietal lobe may serve as a predictive indicator for assessing the likelihood of consciousness improvement in patients diagnosed with VS/UWS.

The nonlinear indicator, SamEn, exhibits limited diagnostic value in DOC. The SamEn values were significantly lower in patients with VS/UWS in contrast to those with higher levels of consciousness. SamEn quantifies time series complexity by demonstrating the potential of a nonlinear dynamical system to produce novel patterns [41]. It is simpler to compute and primarily serves to quantitatively depict system complexity and regularity. However, the diagnostic validity of PE, which is also a nonlinear indicator, was higher than that of SamEn in the present study. This may be attributed to the higher stability and greater noise immunity of PE. This is reinforced by the fact that the EEG data analyzed in the present study had a limited duration of only 30 min and a relatively short time series.

The results of this study indicate that QPC and QPSC do not play a significant role in the diagnosis and prognostic assessment of DOC. In individual comparisons, the diagnostic significance of QPSC classification is limited. Specifically, δ-QPSC values in VS/UWS patients were significantly higher than those in MCS patients in the P4 lead. θ-QPSC values in VS/UWS patients were significantly higher than those in EMCS patients in both P3 and P4 leads, and were significantly higher than those in MCS patients in the P4 lead. α-QPSC values in EMCS and MCS patients were significantly higher than those in VS/UWS patients in both F3 and P4 leads. EMCS and MCS patients exhibited significantly lower θ-α QPC values in the P3 lead compared to VS/UWS patients, indicating that QPC in the parietal lead can serve as an objective reference indicator for DOC classification diagnosis. Previous studies have indicated that θ-QPSC values can effectively distinguish between patients with VS/UWS and those with MCS [51,52,53]. This study suggests that the θ-QPSC values at the parietal leads hold greater diagnostic value. Additionally, δ-QPSC and α-QPSC could also offer diagnostic insights for VS/UWS. Nevertheless, none of these parameters demonstrate significant diagnostic utility within the same individual. The discrepancy may stem from the fact that QPSC, being a power-independent bi-spectral approach, assesses the interactions among distinct neural oscillations, thereby revealing the inherent coupling of neural activities [25]. In contrast, variations in the intrinsic coupling of neural activities within the same in vivo setting are not as pronounced as those observed between individuals. The findings regarding the QPC of θ-α indicator align with prior research [51,52,53], indicating a strong association between QPC of θ-α and the depth of anesthesia or level of consciousness in patients. The study found that the patients with VS/UWS did not exhibit significant variations in QPC across all leads. Additionally, the diagnostic efficacy of both QPC and QPSC was not deemed substantial for patients with differing levels of consciousness. In this study, it was found that patients diagnosed with VS/UWS had a significant decrease in the SI of bedside rs-EEG acquisitions, both within individual patients and across different individuals. This finding aligns with prior studies [54], showing a significant alteration in the SI index as a patient transition from wakefulness to a comatose state and eventually to recovery. Moreover, the SI appears to exhibit greater resilience to noise in patients under anesthesia. The current study revealed that differences in SI among individuals with VS/UWS were specifically evident in the parietal lobe, mirroring QPC. This phenomenon may be associated with diverse neural activities and neurophysiological mechanisms in the parietal lobe [55], which are expected to play a significant role in the restoration of consciousness.

The wPLI index, a bedside EEG feature indicating functional connectivity, does not significantly contribute to the categorical diagnosis of DOC, showing distinctions primarily between individuals with MCS and those with EMCS. The wPLI at F4−P4 exhibited a lower value in patients with MCS compared to those with EMCS, suggesting a potential association between the strength of frontal−parietal connectivity and the level of consciousness. In contrast, the wPLI demonstrated effectiveness in prognosticating the potential for improved consciousness in patients with DOC. The wPLI-β levels in the frontal lobe leads of patients in VS/UWS (improved) were notably elevated compared to in VS/UWS (unimproved). Similarly, the wPLI-α levels in the F4−P4 region of patients in MCS (improved) were significantly higher than in MCS (unimproved). The findings indicate that the wPLI-β in frontal lobe may serve as a predictive indicator for the probability of VS/UWS (improved). Moreover, the wPLI-α at F4−P4 could potentially function as a predictor for enhanced consciousness in individuals diagnosed with MCS. The connectivity variations were present between patients with favorable and unfavorable prognoses after 24 h of being in a coma [27]. In patients with VS/UWS, functional networks exhibited reduced integration, while the global efficiency attributes of the wPLI networks in both θ and α bands were notably higher in MCS compared to in VS/UWS [56]. The variance in wPLI spectra for forecasting patient prognosis across varying levels of consciousness may stem from distinct levels of phase synchronization between specific brain regions during the initial stages of patients with differing consciousness levels. This discrepancy is further compounded by varying degrees of consciousness enhancement and the wide range of enhancement levels, resulting in notable fluctuations in wPLI values. With the restoration of the default mode network, the phase lag in information transmission between different regions within the brain network shows gradual enhancement [57].

Compared to linear measures like PSD analysis—which primarily capture rhythmic activity but often fail to characterize complexity in the predominantly non-rhythmic, low-frequency EEG of DOC patients—PE provides a more comprehensive assessment of neurodynamic complexity. Unlike functional connectivity metrics such as the wPLI that evaluate inter-regional synchronization and network-level integration, PE focuses on local information processing capacity, offering a complementary perspective on brain function. These distinct yet complementary dimensions together provide a more complete picture of neural states in DOC. PE also offers practical advantages for bedside monitoring: It is computationally efficient, robust to artifacts, does not require long recording durations and is readily implementable on portable devices—making it well suited for real-time clinical applications. Beyond diagnostic utility, PE demonstrated prognostic value in our study. VS/UWS patients who later showed consciousness improvement exhibited significantly higher parietal PE values at baseline compared to those without improvement, suggesting that parietal PE may serve as a predictive marker for consciousness recovery in this population. This regional specificity aligns with the parietal lobe’s established role in conscious awareness as part of the default mode network. PE can serve as an objective adjunct to behavioral assessments such as the Coma Recovery Scale-Revised (CRS-R), particularly valuable in patients unable to cooperate due to motor deficits or fluctuating awareness, and for capturing dynamic consciousness changes during continuous monitoring. Future research should establish optimal PE cutoff values for discriminating consciousness levels (e.g., VS/UWS vs. MCS) and predicting recovery using large, multicenter datasets with receiver operating characteristic analysis. Individualized thresholds accounting for etiology, disease duration, and age may further enhance diagnostic precision. Integrating PE with connectivity measures (e.g., wPLI) and neuroimaging modalities holds promise for developing a multidimensional assessment framework to improve management and prognostication in DOC. From the perspective of modern neural oscillation theory, the dominance of slow waves reflects thalamocortical disconnection and impaired integrative processing, while the maintenance of α- and β-band synchrony supports the large-scale intracerebral communication required for consciousness. The association we observed between high displacement entropy, elevated α/β-band wPLI values, and improved behavioral measures of consciousness is generally consistent with these theoretical frameworks. However, scalp rs-EEG signals also reflect overall arousal levels and diffuse cerebral dysfunction. Therefore, these biomarkers correlate with behaviorally measured indicators of consciousness rather than serving as direct measures of subjective, internal conscious experience.

Several limitations of this study should be acknowledged. First, prognosis was assessed merely based on changes in consciousness level, without considering survival and functional rehabilitation, which may not capture comprehensive clinical outcomes. Second, only four-channel rs-EEG covering frontal−parietal regions was adopted, limiting spatial resolution and whole-brain network characterization, while ongoing 128 channel EEG research may compensate for this shortcoming. Third, this cohort included DOC patients with heterogeneous etiologies. Although baseline characteristics were balanced across subgroups, etiology stratified analyses of EEG biomarkers were not performed. Uncontrolled retrospective confounders such as sedative exposure and ventilation duration may also interfere with EEG signals. Fourth, the absence of healthy controls hinders the definition of normative EEG reference values. Fifth, patients with skull defects were not systematically excluded, which might bias EEG connectivity-related measurements. In addition, we cannot completely dissociate EEG alterations specific to consciousness impairment from signals reflecting deficits in arousal, attention, and other cognitive domains secondary to diffuse brain injury. Furthermore, the imbalanced sample size between the improvement group (n = 54) and non-improvement group (n = 153) may lower statistical power and introduce random fluctuations, as consciousness improvement is an infrequent clinical event in DOC populations. Finally, machine learning-based classification models were not constructed. Future multimodal, large sample prospective studies with high-density EEG, matched healthy controls, and neuropsychological evaluation are required to further validate these biomarkers.

In summary, although our study demonstrates the potential of bedside rs-EEG indices (particularly PE and wPLI) for evaluating consciousness levels and predicting recovery in DOC patients, the above limitations should be addressed in future multicenter and large-sample validation studies.

## 5. Conclusions

In conclusion, this study demonstrates that bedside rs-EEG serves as a valuable, non-invasive tool for objectively evaluating brain function in DOC patients with severe motor limitations. Owing to its convenient acquisition and clinical feasibility, rs-EEG is particularly suitable for bedside dynamic monitoring. Among the rs-EEG features analyzed in this study, PE provides robust and valuable information for differentiating various consciousness levels and assisting DOC classification. Furthermore, our results indicate that parietal PE, as well as wPLI values within the β and α frequency bands, is closely correlated with neurological improvement in DOC patients. These EEG markers may reflect the functional recovery potential of residual brain activity and are suggestive of better consciousness outcomes. However, these associations should not be interpreted as precise predictive performance. Future large-sample studies with quantitative prognostic modeling are required to validate their exact predictive efficacy.

## Figures and Tables

**Table 1:** Comparison of demographic characteristics of DOC patients.

Demographic Characteristics	Unimproved Group (n = 161)			Improved Group (n = 54)			Total (n = 215)	H / Z / $X^{2}$	*p*
	VS/UWS (n = 85)	MCS (n = 68)	EMCS (n = 8)	MCS (from VS/UWS) (n = 27)	EMCS (from VS/UWS) (n = 11)	EMCS (from MCS) (n = 16)			
age (years)	57 (47, 68)	58 (48.5, 66)	42 (19.75, 59.75)	57 (50, 65)	46 (36, 66)	59 (44.25, 62.5)	57 (46, 66)	H = 4.739	0.449
course of disease (days)	48 (32, 75)	37 (25, 63)	38 (13, 47)	42 (24, 73)	42 (35, 60)	36 (27, 51)	41 (28, 66)	Z = −0.501	0.622
genders (%)								$X^{2}$ = 0.07	0.793
male	45 (35.00)	47 (37.00)	3 (2.00)	16 (13.00)	8 (6.00)	9 (7.00)	128 (60.00)		
female	40 (46.00)	21 (24.00)	5 (6.00)	11 (13.00)	3 (3.00)	7 (8.00)	87 (40.00)		
cause of disease (%)								$X^{2}$ = 6.43	0.091
TBI	34 (37.00)	22 (24.00)	5 (6.00)	14 (15.00)	5 (6.00)	11 (12.00)	91 (42.00)		
strokes	41 (38.00)	43 (40.00)	2 (2.00)	11 (10.00)	6 (5.00)	5 (5.00)	108 (50.00)		
HIE	7 (70.00)	1 (10.00)	0 (0.00)	2 (20.00)	0 (0.00)	0 (0.00)	10 (5.00)		
others	3 (50.00)	2 (33.00)	1 (17.00)	0 (0.00)	0 (0.00)	0 (0.00)	6 (3.00)		
injured side (%)								$X^{2}$ = 1.49	0.224
left	36 (34.00)	35 (34.00)	3 (3.00)	16 (15.00)	7 (7.00)	7 (7.00)	104 (48.00)		
right	49 (44.00)	33 (30.00)	5 (4.00)	11 (10.00)	4 (4.00)	9 (8.00)	111 (52.00)		

## Data Availability

The data supporting the findings of this study are not publicly available due to privacy and ethical restrictions. De-identified data may be made available by the corresponding author upon reasonable request and subject to approval by the relevant institution-al ethics committee.

## Abbreviations

The following abbreviations are used in this manuscript:

DOC	disorders of consciousness
rs-EEG	resting-state electroencephalography
VS/UWS	vegetative state/unresponsive wakefulness syndrome
MCS	minimally conscious state
EMCS	emergence from the minimally conscious state
CRS-R	Coma Recovery Scale-Revised
EEG	electroencephalographic
RBP	relative band power
PSD	power spectral density
QPC	quadratic phase coupling
QPSC	quadratic phase self-coupling
GHWT	general harmonic wavelet transform
PE	permutation entropy
SamEn	Sample Entropy
SI	synchronization index
wPLI	weighted Phase Lag Index
